# Atomically Dispersed Palladium Promoted Suzuki–Miyaura Cross Coupling

**DOI:** 10.1002/cssc.202500953

**Published:** 2025-09-10

**Authors:** Junhao Huang, Marcus Klahn, Stephan Bartling, Anna Zimina, Nils Rockstroh, Norbert Steinfeldt, Tim Peppel, Jan‐Dierk Grunwaldt, Jennifer Strunk

**Affiliations:** ^1^ Leibniz Institute for Catalysis e.V. Albert‐Einstein‐Straße 29a 18059 Rostock Germany; ^2^ Institute of Catalysis Research and Technology and Institute for Chemical Technology and Polymer Chemistry Karlsruhe Institute of Technology (KIT) 76131 Karlsruhe Germany; ^3^ Industrial Chemistry and Heterogeneous Catalysis Technical University of Munich Lichtenbergstraße 4 85748 Garching Germany

**Keywords:** attenuated total reflection‐Fourier transform infrared spectroscopy, heterogeneous Pd catalysts, polymeric carbon nitride, Suzuki coupling

## Abstract

The palladium‐catalyzed Suzuki**–**Miyaura cross coupling reaction to forge carbon‐carbon bonds fundamentally changes the practice of organic synthesis. Herein an isolated palladium catalyst supported on polymeric carbon nitride (Pd/PCN) for efficient cross coupling of bromobenzene and phenylboronic acid at room temperature is reported. It is demonstrated that the Pd/PCN catalyst with a 2 wt% Pd loading achieves the highest mole‐specific activity. In addition, the size of supported Pd can strongly affect the reaction performance: the isolated Pd species exhibit higher activity compared to the Pd nanoparticles. The continuous flow tests demonstrate that the catalytic properties of the Pd/PCN catalyst strongly depend on the reaction atmosphere: Pd‐catalyzed self‐coupling of phenylboronic acid as a side reaction is more pronounced under an O_2_ flow than in an Ar flow. Detailed mechanistic investigations through in situ infrared spectroscopy reveal the role of the base K_2_CO_3_ in activating the phenylboronic acid.

## Graphical abstract

An isolated palladium catalyst supported on polymeric carbon nitride is prepared and applied for the Suzuki‐coupling reaction under ligand‐free conditions at room temperature. Moreover, in situ infrared spectroscopy reveals the role of the base K_2_CO_3_ and characterizes the dynamic changes in substrate consumption and product formation during the cross‐coupling process.

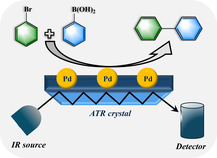

## 1 Introduction

The Suzuki**–**Miyaura reaction is a key step in the total synthesis of natural products and pharmaceuticals.^[^ ^1^ ^]^ To date, palladium remains the most effective metal for driving this reaction.^[^ ^2^ ^]^ Substantial progress has been made in recent years in the development and implementation of heterogeneous Pd catalysts.^[^ ^3^ ^]^ For example, the synthesis of Sitagliptin, an active pharmaceutical ingredient used to treat type 2 diabetes, involves a large‐scale Suzuki coupling reaction using heterogeneous Pd/C as the catalyst.^[^ ^4^ ^]^ This synthesis is ligand‐free, which helps minimize costs and simplify the purification process. However, the high Pd loading required for Pd nanoparticle catalysts raises concerns about the sustainability of such approaches, particularly as the economically accessible Pd resources continue to decline.^[^ ^5^ ^]^ One potential strategy to address this challenge is to enhance atomic utilization efficiency by reducing the size of the supported metal.

The fast development of synthetic chemistry and characterization techniques has enabled the design of heterogeneous catalysts at the molecular and even atomic levels.^[^ ^6^ ^]^ The isolated Pd catalysts as newcomer catalysts in this family offer superior and cost‐effective solutions.^[^ ^7^ ^]^ So far, various inorganic and organic materials have been adopted to stabilize the atomic Pd species, such as CeO_2_,^[^ ^8^ ^]^ TiO_2_,^[^ ^9^ ^]^ metal‐organic framework (MOF),^[^ ^10^ ^]^ metal‐organic polyhedra (MOP),^[^ ^11^ ^]^ polymeric carbon nitride (PCN),^[^ ^12^ ^]^ and N‐doped carbon nanosheets.^[^ ^13^ ^]^ As a representative example, Chen et al. have reported on the application of 0.66 wt% isolated Pd/PCN catalyst for the flow Suzuki**–**Miyaura cross coupling reactions in the presence of triphenylphosphine (PPh_3_) ligand, which outperformed both homogeneous Pd complexes and heterogeneous Pd nanoparticles.^[^ ^12^ ^]^ However, further increasing the Pd loading to 1.25% resulted in a decline in activity, attributed to the formation of Pd clusters. In this case, the growth of Pd size diminishes catalytic performance. Thus, exploration of an effective strategy to enhance Pd loading while maintaining Pd in form of isolated sites is highly needed. Recently, our group has reported on a catalyst system containing 2 wt% isolated Pd atoms on PCN successfully prepared by applying a facile wet impregnation method.^[^ ^14^ ^]^ Combining surface science techniques with theoretical calculations, we have demonstrated that Pd atoms were stabilized on the carbon nitride support via robust Pd—N bonds. The catalysts thus prepared are also anticipated to be effective in cross coupling reactions.

Notably, since the catalytic site is a single, isolated atom, the Pd site can serve as a well‐defined platform for understanding the reaction mechanism. Such studies are often conducted in combination with advanced characterization techniques. Multiple analytical methods have been applied for the identification of reaction intermediates, such as quick‐scanning extended X‐ray absorption fine structure (QEXAFS) spectroscopy,^[^ ^15^ ^]^ rapid injection NMR (RI‐NMR),^[^ ^16^ ^]^ and electrospray ionization mass spectrometry (ESI‐MS) (see Supplementary Note for details).^[^ ^17^ ^]^ Recent advances in attenuated total reflection (ATR)‐Fourier transform infrared (FTIR) spectroscopy, along with the development of specialized in situ cells, have proven valuable in elucidating the process of organic transformations.^[^ ^18^ ^]^ Importantly, ATR‐FTIR spectroscopy is a non‐invasive, non‐destructive analytical technique, and enables liquid samples to be analyzed in situ.^[^ ^19^ ^]^ Accordingly, ATR‐FTIR spectroscopy is well‐suited for investigating the reaction mechanism of the Suzuki coupling reaction over isolated Pd species.

In this work, we present a 2 wt% isolated Pd/PCN catalyst utilized for catalyzing Suzuki**–**Miyaura cross coupling reaction at room temperature without the use of ligands. The effect of reaction atmosphere (oxygen or argon) on the catalytic properties of atomically Pd is investigated. In addition, we apply high temperature calcination methods to get larger Pd clusters or even nanoparticles to evaluate the influence of Pd particle size on the catalytic performance. The potential enhancement of the photothermal effect through visible‐light irradiation on reaction efficiency is also explored. Furthermore, by using a comprehensive analysis by X‐ray absorption spectroscopy (XAS) including wavelet transform and related methods, we have monitored the fate of the catalysts. Finally, ATR‐FTIR spectroscopy was used to probe the role of the base K_2_CO_3_ in the formation of reactive boronate intermediates.

## 2 Results and Discussion

**Figure 1:**
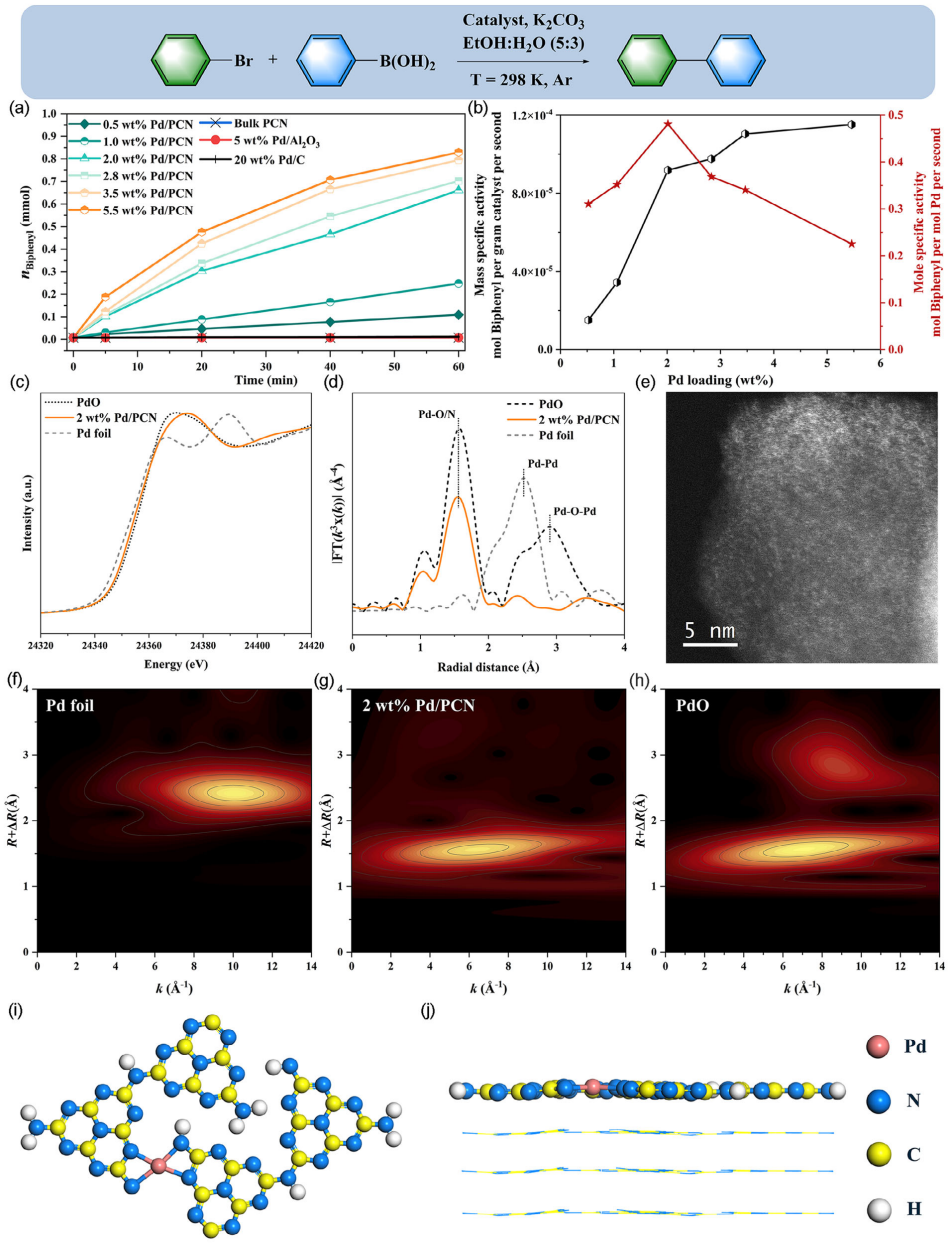
a) Biphenyl formation over bulk PCN, Pd/PCN catalysts, commercial 5% Pd/Al_2_O_3_, and commercial 20 wt% Pd/C. Reaction conditions can be found in experimental section. b) The mole specific activity (mol Biphenyl per mol Pd per second, right axis, red) and mass specific activity (mol Biphenyl per unit mass of the catalyst per second, left axis, black) of the various Pd/PCN catalysts. c) Pd K‐edge XANES spectra, d) corresponding Fourier transformed EXAFS spectra (FT‐EXAFS, not corrected for the phase shift, k^3^‐weighted), and e) STEM‐HAADF image for 2.0 wt% Pd/PCN. Wavelet transform analysis of Pd K‐edge EXAFS oscillations of f) Pd foil, g) 2 wt% Pd/PCN, and h) PdO. i) Top view and j) side view of the DFT‐optimized structure models for 2.0 wt% Pd/.PCN.^[^ ^40^ ^]^

Pd/PCN catalysts with the target loading of 0.5, 1.0, 2.0, 4.0, 8.0, and 16.0 wt% were prepared via a wet impregnation method, followed by thorough washing with acetone to remove loosely bound Pd salts (see Experimental Section for detail). The actual Pd loadings in the resultant catalysts were 0.5, 1.0, 2.0, 2.8, 3.5, and 5.5 wt%, respectively, as confirmed by inductively coupled plasma‐optical emission spectroscopy (ICP‐OES). We evaluated the reactivity of various Pd/PCN catalysts using a typical cross coupling reaction between bromobenzene (PhBr) and phenylboronic acid [PhB(OH)_2_] in a batch reactor, conducted without the addition of ligands. As shown in **Figure** **1**a, pristine PCN, commercial 5% Pd/Al_2_O_3_, and commercial 20% Pd/C catalysts were all inactive at 25 °C. For the Pd/PCN catalysts, the yield of biphenyl drastically increased when the Pd loading was 2.0 wt%, showing the highest mole specific activity (Figure 1b and Table S1, Supporting Information). Analysis of the 2.0 wt% Pd/PCN sample by extended X‐ray absorption fine structure (EXAFS) spectra confirmed that the supported Pd species were mononuclear due to the absence of backscattering from higher shells (Fourier‐transformed EXAFS, Figure 1d and Table S2, Supporting Information). EXAFS, e.g., did not evidence any Pd−Pd bond. The wavelet transform (WT) EXAFS contour plot of 2.0 wt% Pd/PCN displayed only one intensity maximum at 6.3 Å^−1^ in contrast to Pd and PdO references, attributed to the backscattering between Pd and light atoms (Figure 1f–h).[^6^, ^20^] Further STEM investigations (Figure 1e) proved that Pd sites were atomically dispersed on the PCN surface, with an only negligible number of Pd nanoparticles (2–3 nm). The normalized X‐ray absorption near edge structure (XANES) spectra for the Pd/PCN (Figure 1c), along with the reference Pd foil, suggest a positively charged state of Pd species induced by electron transfer from Pd to the support.^[^ ^21^ ^]^ Additionally, the Pd/PCN sample exhibits a shift of the white line at 24 370 eV towards higher energy relative to the PdO spectra, which is attributed to the formation of Pd—N coordination. The EXAFS‐derived coordination number of 4 indicates that each Pd atom is, on average, bonded to four surface nitrogen atoms with an average distance of 2.04 Å (Table S2, Supporting Information). The atomic structure model was illustrated in Figure 1i,j. In comparison, the 2.8, 3.5, and 5.5 wt% Pd/PCN catalysts exhibited a considerable decline in mole‐specific activity (Figure 1b), attributed to the formation of Pd clusters as confirmed in a previous work.^[^ ^14^ ^]^ Although both the isolated Pd atoms and exposed Pd atoms on the surface of clusters are expected to contribute to the overall activity, Pd atoms in the interior of the clusters are not accessible to reactant molecules and cannot be directly involved in the catalytic process.

**Figure 2:**
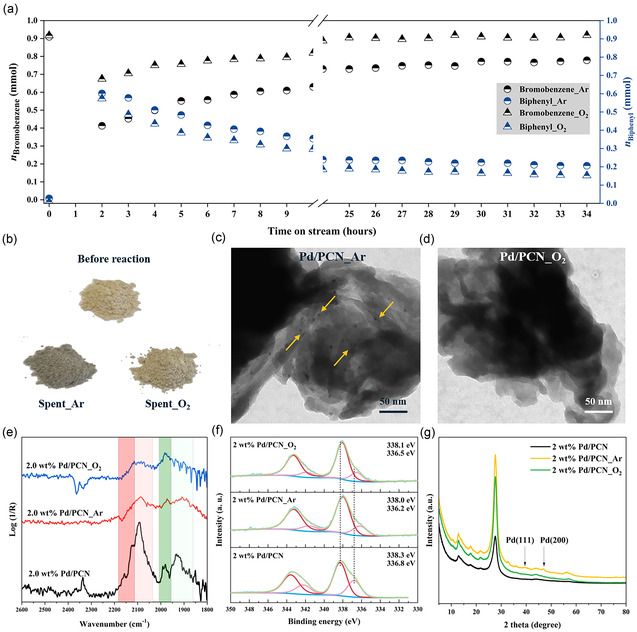
a) Continuous flow experiments of Suzuki cross coupling of PhBr with PhB(OH)_2_ over 2.0 wt% Pd/PCN in Ar and O_2_. b) Sample photographs. Representative TEM images of spent catalysts 2.0 wt% Pd/PCN_Ar c) and 2.0 wt% Pd/PCN_O_2_ d). e) CO‐DRIFTS spectra, f) Pd 3 d X‐ray photoelectron spectra, and g) XRD patterns for 2.0 wt% Pd/PCN, 2.0 wt% Pd/PCN_Ar, and 2.0 wt% Pd/PCN_O_2_.

We further evaluated the 2.0 wt% Pd/PCN sample in a continuous flow reactor. The solution concentration utilized in the continuous flow test was consistent with that used in the batch mode. As shown in **Figure** **2**a, biphenyl is formed in both Ar and O_2_ atmospheres, but the catalyst exhibited atmosphere‐dependent behaviors. Specifically, in an O_2_ atmosphere, after 29 h, 0.17 mmol biphenyl were produced, but bromobenzene had almost no consumption. This indicates that the biphenyl was produced solely via the Pd‐catalyzed self‐coupling reaction of phenylboronic acid. The contribution from the cross coupling reaction is not significant. Conversely, under an Ar atmosphere after 29 h, 0.16 mmol of bromobenzene was consumed, while 0.20 mmol of biphenyl was generated, indicating that 0.04 mmol of biphenyl resulted from the side reaction. This observation suggests that both the cross coupling of PhBr and PhB(OH)_2_ and the self‐coupling of PhB(OH)_2_ occurred under Ar condition. This finding was supported by high‐performance liquid chromatography (HPLC) data as presented in Table S3, Supporting Information, which showed strong cross coupling and weak self‐coupling under Ar conditions. Conversely, under O_2_ conditions, weak cross coupling and strong self‐coupling were observed. There are two potential explanations for these atmosphere‐dependent properties. The first is that O_2_ disrupts the Pd—N bond, resulting in a weaker coordination structure of Pd to the support. Consequently, the spatial and electronic structures of Pd are changed, facilitating the self‐coupling of two PhB(OH)_2_ molecules on the palladium. The second explanation is that the low‐charge‐state Pd species was oxidized by O_2_ to form high‐charge‐state of Pd species, which can then facilitate the aryl transfer from PhB(OH)_2_.

However, catalyst deactivation was observed over time on stream (Figure 2a and Table S4, Supporting Information). Meanwhile, we found that the color of the Ar‐spent sample changed to dark gray (Figure 2b). The spent samples were then thoroughly characterized by XPS, CO‐DRIFTS, XRD, UV‐Vis DRS, and TEM. The CO‐DRIFTS spectra (Figure 2e) show that the band intensities of the linear CO on isolated Pd cations at 2123 and 2090 cm^−1^ are not as pronounced as in the fresh samples. The Pd 3 d XPS exhibited slightly negative shifts in binding energies for both Ar‐ and O_2_‐spent catalysts (Figure 2f), with the Ar‐spent catalyst showing a greater shift than the O_2_‐spent catalyst. These changes in CO‐DRIFTS and XPS suggest the aggregation of surface Pd species during the reaction. This finding was further supported by XRD, where reflections centered at 39.6° and 47.1°, associated with Pd (111) and Pd (200), were found in the Ar‐spent sample, confirming the presence of Pd nanoparticles (Figure 2g).^[^ ^22^ ^]^ High‐resolution TEM images (Figure 2c,d) provide straightforward evidence of Pd nanoparticles being formed in the Ar‐spent sample, while much fewer Pd nanoparticles were observed in the O_2_‐spent sample, indicating that Pd aggregation is more severe in an Ar atmosphere than in an O_2_ atmosphere. It is possible that oxygen can adhere to the surface of Pd, particularly in oxygen‐rich environments, and cause in situ oxidation of Pd, thereby leading to minimal change in the Pd charge state as detected by XPS.^[^ ^23^ ^]^ Notably, the Pd content remained almost unchanged before and after the reaction (ICP‐OES, Table S5, Supporting Information), excluding an activity decay caused by a massive loss of Pd content. Additionally, the reaction filtrates from both batch and continuous flow measurements were analyzed by ICP‐OES. No detectable Pd was observed in the liquid phase, suggesting that no significant amount of Pd leaching occurred. This result is consistent with the negligible change in Pd content in the spent catalysts. Furthermore, a high concentration of potassium (K) was observed in the spent catalysts, as shown in XPS and ICP analysis (Table S5, Supporting Information). Therefore, the reasons for the deactivation might be the sintering of Pd, the aggregation of Pd and K or both. Notably, a dynamic leaching‐redeposition cycle is a possible mechanism for the aggregation of Pd nanoparticles.^[^ ^15^, ^24^ ^]^ Since Pd single atoms are highly active during the reaction, surface Pd species can exhibit dynamic behavior and migrate, eventually aggregating into nanoparticles. This dynamic movement may also allow Pd atoms to enter the solution, this process referred to as Pd leaching. However, since the overall Pd content does not significantly change, and Pd was not detected in the solution, a redeposition process must also occur. Based on the insight gained so far, a mechanism comprising leaching immediately followed by redeposition cannot be clearly distinguished from simple Pd migration along the surface.

**Figure 3:**
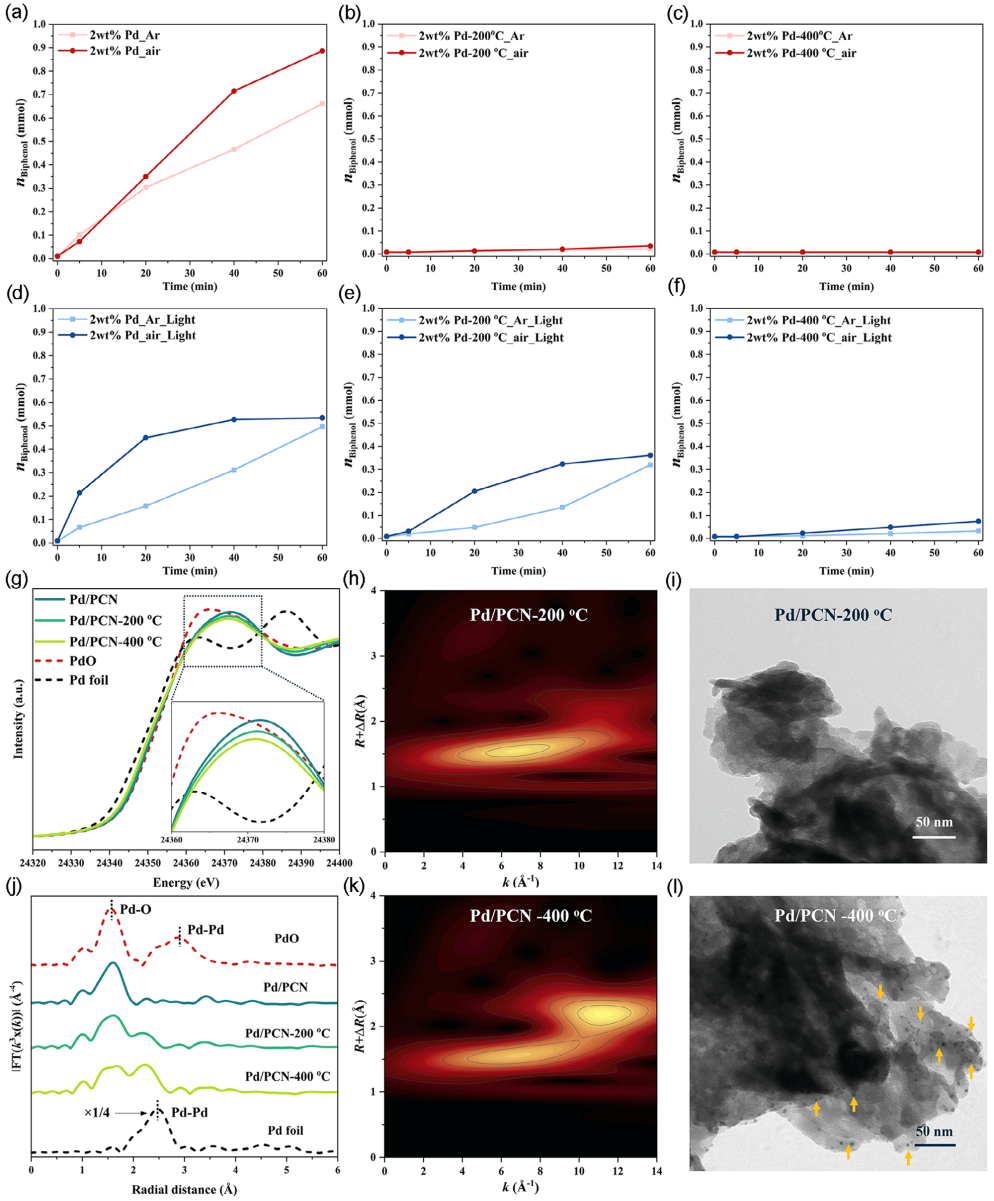
Biphenyl formation using various Pd catalysts in argon or air: a) 2.0 wt% Pd/PCN, b) 2.0 wt% Pd/PCN‐200 °C, and c) 2.0 wt% Pd/PCN‐400 °C. The same catalysts under light irradiation: d) 2.0 wt% Pd/PCN_light, e) 2.0 wt% Pd/PCN‐200 °C_light, and f) 2.0 wt% Pd/PCN‐400 °C_light. g) Pd K‐edge XANES spectra and j) EXAFS magnitude of the Fourier transform. Wavelet transform analysis of Pd K‐edge EXAFS oscillations of h) 2.0 wt% Pd/PCN‐200 °C and k) 2.0 wt% Pd/PCN‐400 °C. Representative TEM images of i) 2.0 wt% Pd/PCN‐200 °C and l) 2.0 wt% Pd/PCN‐400 °C.

We then studied the effect of Pd particle size on catalytic performance. The 2 wt% Pd/PCN sample was calcined at 200 and 400 °C, respectively, and the performance was subsequently tested in the batch mode. However, an almost complete suppression of activity was observed after high‐temperature calcination for 2 wt% Pd/PCN, regardless of the reaction atmospheres (**Figure** **3**a–c and Table S6, Supporting Information). XANES analysis (Figure 3 g) revealed that the white‐line intensity decreased as the calcination temperature increased, suggesting a decrease in Pd oxidation state. Simultaneously, the FT‐EXAFS spectra showed an increase in the backscattering at 2.2 Å (Figure 3j) with the change of calcination temperature. This peak is attributed to the Pd backscattering, indicating the existence of the Pd nanoparticles. Further WT contour plot (Figure 3h,k) demonstrated the Pd−Pd interaction by the presence of a second‐shell intensity maximum at 11.3 Å^−1^. These findings correlate well with XRD, XPS, and CO‐DRIFTS analyses reported in our previous work.^[^ ^14^ ^]^ Additionally, the TEM imaging studies (Figure 3i,l) revealed that a significant number of Pd nanoparticles was formed in the 400 °C calcined sample, but only very few were observed in the 200 °C calcined sample. Figure S3, Supporting Information, showed that Pd retained mostly in isolated sites after calcination at 200 °C, while a 3–4 nm nanoparticle is also observed. This small nanoparticle exhibited a shorter Pd—Pd bond distance of 2.45 Å than the 2.74 Å in the Pd foil attributed to the effect of metal‐support interaction (Table S2, Supporting Information).^[^ ^25^ ^]^ More importantly, the above characterization results suggest that isolated Pd atoms are initially incorporated into the PCN network before calcination. After calcination at 200 °C, the connection between the Pd atom and the PCN network becomes less defined; in other words, the Pd–N bonds weaken, possibly allowing Pd atoms to detach from the PCN sheet and start aggregating. At 400 °C, the PCN network is further destroyed, leading to Pd migration and aggregation into large nanoparticles. Therefore, the decline in performance can be attributed to the growth of the Pd particle size.

The high activity of isolated Pd atoms on PCN at 25 °C in the dark is a favorable result. However, the system can also be considered a potential photocatalyst, given that PCN is a semiconductor, and Pd can function as active site. PCN can be activated upon visible light irradiation, generating electrons that are transferred to the Pd center, thereby potentially facilitating the reaction.^[26]^ Thus, the question whether irradiation can increase the activity is intriguing. However, light did not contribute to the reactivity of the uncalcined sample and even decreased it (Figure 3d). In the early stages of the reaction, it appears that light has a beneficial effect if the reaction is carried out in air, but eventually, the sample deactivates quickly. When run in argon atmosphere, the photoreaction is worse than the dark reaction over the whole reaction time. This can be attributed to the aggregation of Pd atoms into larger nanoparticles caused by the photoreduction effect, which is also a well‐known method for photo‐deposition of metal nanoparticles onto support surfaces.^[^ ^27^ ^]^ Such an effect is likely faster in argon compared to air, because oxygen might potentially compete with the metal for photogenerated electrons, so superoxide might be formed.

In the calcined Pd/PCN samples, where the formation of biphenyl is fully suppressed in the absence of light, some of the activity can be restored under irradiation (Figure 3e,f). A higher yield of biphenyl was consistently observed in O_2_ than in Ar. This could be attributed to the occurrence of the self‐coupling of PhB(OH)_2_ in O_2_ as side reaction, as observed in the continuous flow test.

**Figure 4:**
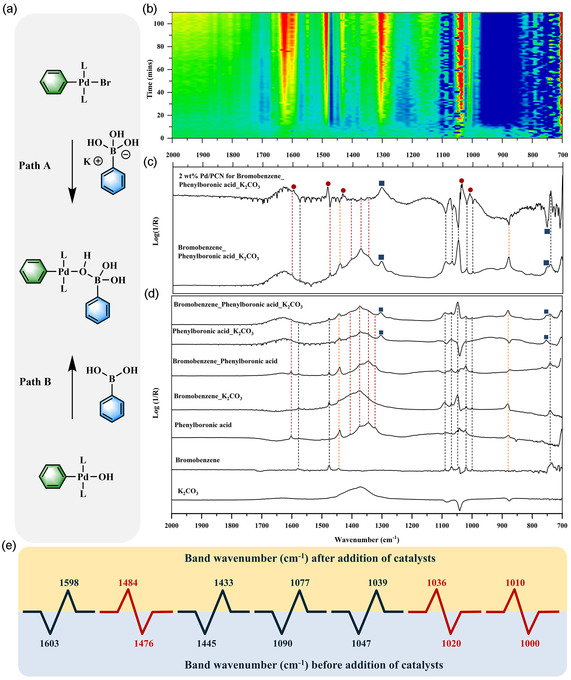
a) Proposed transmetalation pathways in the Suzuki–Miyaura process. b) Time series 2D ATR‐IR spectra and c) typical ATR‐FTIR spectra of 2 wt% Pd/PCN for the coupling reaction of PhBr and PhB(OH)_2_. In the 2D IR spectra, negative bands are shown in blue and positive bands in red. d) ATR‐FTIR spectra of the substrate calibration (PhBr, PhB(OH)_2_, and K_2_CO_3_). The bands related to the functional groups from PhBr and PhB(OH)_2_ are marked with black and red dashed lines, respectively. The yellow dashed lines mark the IR bands present in both substrates. Newly emerged bands derived from boron‐related groups are marked with dark blue squares, and new bands related to biphenyl are marked with dark red spheres. e) Shift in band frequencies of the functional groups in the substrates before and after addition of the catalyst.

In situ ATR‐FTIR spectroscopy was performed to gain insight into the reaction mechanism. To date, one general consensus regarding the Suzuki**–**Miyaura reaction is that the catalytic cycle follows a sequence involving (i) oxidative addition of an aryl halide to a Pd site to form an arylpalladium intermediate, (ii) transmetalation with a boronic acid, and (iii) reductive elimination from the resulting diarylpalladium intermediate to afford the corresponding biaryl.^[^ ^28^ ^]^ Importantly, a base is required for this reaction. However, the precise role of base is still under debate.^[^ ^29^ ^]^ According to the reported computational, spectroscopic, and kinetic studies,^[^ ^30^ ^]^ two roles are considered: one is that the base facilitates the slow transmetalation of the boronic acid by forming a more reactive boronate species that can interact with the Pd center and transmetalate in an intramolecular fashion (**Figure** **4**, Path A).^[^ ^31^ ^]^ The alternative is that the base replaces the halide in the coordination sphere of the palladium complex and facilitates an intramolecular transmetalation (Figure 4, Path B). At this point, a key question that remains is whether the boron species will change after mixing with the base.

First, in the step of substrate calibration (without addition of catalysts), after mixing the PhB(OH)_2_ with K_2_CO_3_, two new bands at 1304 and 752 cm^−1^ emerged, attributed to the B−O stretching and C−H out‐of‐plane vibration (Figure 4d and Table S7, Supporting Information).^[^ ^32^ ^]^ It is known that the predominant species in solution of an aqueous solution of boric acid [B(OH)_3_] with pH > pKa is ionized tetra‐coordinated B(OH)_4_^−^.^[^ ^33^ ^]^ Thus, it is reasonable to speculate that phenylboronic acid transforms into a tetrahedral boronate complex at high pH after addition of K_2_CO_3_. The formed complex is referred to as a negatively charged aryltrihydroxyboronate.^[^ ^16^ ^]^ Importantly, the appearance of new infrared bands demonstrates the interaction between K_2_CO_3_ and PhB(OH)_2_. Further evidence of the effect of base on PhB(OH)_2_ was obtained from ^11^B and ^13^C NMR spectroscopy. The ^11^B NMR signal of PhB(OH)_2_ at 28 ppm shifted slightly upfield to 24 ppm and became broad after mixing with K_2_CO_3_ (Figure S4, Supporting Information). The upfield shift of ^11^B suggests a transformation of a trigonally coordinated boron compound to a tetrahedrally coordinated compound.^[^ ^34^ ^]^ Besides, the ^13^C NMR signals shifted slightly upfield (+0.2 to +0.7 ppm), and the signal at 131 ppm, attributed to the carbon bonded to boron, disappeared (Figure S5, Supporting Information). Therefore, based on the analysis above, we can conclude that the aryl boronic acid is strongly affected by the base, facilitating its conversion into a reactive boronate intermediate.^[^ ^29^, ^31^, ^35^ ^]^

Next, upon addition of the catalyst, the infrared bands associated with the Br−Ph group in bromobenzene at 1069 cm^−1^ turned negative (Figure 4c, Figure S7, and Table S7, Supporting Information).^[^ ^9^ ^]^ Simultaneously, the IR bands within the 1380–1310 cm^−1^ range, linked to the B—O stretching in phenylboronic acid (Figure S8, Supporting Information), exhibited a significant decrease.^[^ ^32^ ^]^ These spectral changes in bromobenzene and phenylboronic acid suggest the initiation of a cross coupling reaction between the two substrates. Additionally, the enlarged image in Figure S9, Supporting Information, exhibits a newly emerged band at 1042 cm^−1^, possibly attributed to the B−Br stretching.^[^ ^32^ ^]^ Meanwhile, the band intensity at 1304 cm^−1^ keeps increasing over the reaction time, indicating that more B—O groups are formed throughout the reaction process (Figure 4c). Notably, there are shifted frequencies of the aryl group in the product biphenyl (Ar−Ar) compared to the substrates (Ar−Br and Ar−OH) due to changes in the substituent groups. A significant example of this frequency shift is the decrease in band intensity at 1476 cm^−1^ (Figure 4b, blue area) over the reaction time, while a new band at 1484 cm^−1^ (Figure 4b, red area) emerges with its intensity gradually increasing. Similar frequency shifts are depicted in Figure 4e. Thus, we attributed the newly emerged bands at 1598, 1484, 1433, 1077, 1039, 1036, and 1010 cm^−1^ to the functional groups in biphenyl (Figure 4c,e and Figure S10,Supporting Information).^[^ ^36^ ^]^ A similar observation was obtained when measuring another aryl halide, iodobenzene, with phenylboronic acid, again confirming the occurrence of a cross coupling process (Figure S11,Supporting Information).

## 3 Conclusions

We have shown here that a PCN‐supported 2 wt% isolated Pd catalyst effectively catalyzes the Suzuki**–**Miyaura cross coupling reaction at room temperature. The catalytic studies revealed that the reaction behavior of active Pd center is strongly dependent on the reaction atmosphere, with a significant enhancement of self‐coupling reaction observed in an O_2_ atmosphere compared to an Ar atmosphere. Additionally, the reactivity is strongly affected by the Pd particle size, with the isolated Pd sites proving to be highly reactive, whereas the large Pd nanoparticles exhibit complete suppression of activity in the absence of light. ATR‐IR studies highlighted the role of the base K_2_CO_3_ in activating phenylboronic acid, facilitating the formation of a reactive boronate intermediate. The knowledge acquired in this work may inspire further development of palladium‐based heterogeneous catalysts for organic synthesis.

## 4 Experimental Section

4.1

4.1.1

##### Catalyst Preparation

Materials: Urea (99.0−100.5%, Alfa Aesar), Palladium(II) trifluoroacetate (Pd(TFA)_2_, 97%, Sigma‐Aldrich), Acetone (≥ 99.8%, Fisher Chemical), Ethanol (96%, VWR Chemicals), Bromobenzene (1 mmol, 99%, Alfa Aesar), Phenylboronic acid (98+%, Alfa Aesar), and K_2_CO_3_ (≥99%, Carl Roth). Toluene (99.99%, Fisher Chemical). All chemicals were used as received without further purification.

Synthesis of PCN Nanosheet: The PCN nanosheet was prepared according to a reported thermal oxidation exfoliation method.^[^ ^26^ ^]^ Firstly, 15 g of urea was put into a 60 mL ceramic crucible with a cover and heated at 550 °C in a muffle furnace for 4 h in air with a ramp rate of 2 °C min^−1^. Then, the PCN was exfoliated by a thermal oxidation etching method. Specifically, 500 mg of the pristine PCN was placed in a 30 mL ceramic crucible without cover and heated to 500 °C for 3 h in air at a rate of 5 °C min^−1^.

*Synthesis of Pd/PCN Catalysts:* The Pd/PCN catalysts were prepared according to a reported wet impregnation method.^[^ ^14^ ^]^ Five hundred milligrams of PCN nanosheet was dispersed in 25 mL of acetone and stirred for 20 min. Pd(TFA)_2_ was dissolved in acetone to form a 10 mM solution. Then, 4.84 mL of the above solution was added dropwise, with stirring, to the catalyst slurry to achieve the desired 1 wt% loading. The slurry was stirred for another 24 h, and the solvent acetone was gradually removed by evacuation. The powder was then dried at 80 °C for 24 h in Ar flow (100 sccm). The Pd/PCN catalysts with different Pd loadings (0.5, 2, 4, 8, and 16 wt%) were synthesized using the same procedure, with the only variation being the volume of the Pd precursor solution.

To avoid the influence of remaining Pd salts on the Suzuki**–**Miyaura cross coupling, the as‐synthesized Pd/PCN samples were further washed with acetone for six times, and details are as follows: the Pd/PCN samples were first stirred in 300 mL acetone for 30 min then filtered. The same washing and filtration process was repeated three times. Next, the Pd/PCN sample was washed with 300 mL acetone for 3 h, followed by filtration. This procedure was also repeated three times. After that, for samples with a Pd loading greater than 4 wt%, an additional washing with 900 mL of acetone was performed for 3 h, and this was repeated seven times. Sample collection was achieved by filtration with a 0.1 μm PTFE membrane filter. Both the washing and filtering processes are carried out under argon protection at room temperature. The resultant samples were dried in a tube furnace at 80 °C for 12 h in argon flow.

##### Catalytic Tests

Batch Reaction: Coupling of bromobenzene with phenylboronic acid was firstly carried out in batch mode with a 60 mL double jacket cylindrical glass reactor. Before dissolving the substrate, the ethanol and deionized aqueous solutions were processed with a thermal gassing method in argon stream to remove the dissolved oxygen as much as possible. In a typical reaction, the reactor was filled with 40 mL of an ethanol/H_2_O solution (v/v = 5/3) containing bromobenzene (1 mmol), phenylboronic acid (2 mmol), and K_2_CO_3_ (2 mmol). The reactor was evacuated and refilled with Ar by the Schlenk line. This procedure was repeated three times to ensure the complete removal of air from the reactor before the subsequent introduction of catalysts. The reactor was maintained at 298 K. Then, 2 mg of Pd catalyst were added to this reaction mixture. The reaction time is 1 h. During the batch reaction, 1 mL samples were withdrawn from the reactor at specific time intervals; the products were quantified using an Agilent 6890N gas chromatograph, and the internal standard is toluene. For the photocatalytic test, the reaction solution stays the same while being irradiated by a LUMATEC Superlite S04 lamp with a visible light ranging from 400 to 700 nm and an intensity of 225 mW cm^−2^.

Continuous Flow Reaction: The continuous flow operation was performed by using a modular Ehrfeld microreaction system equipped with a cartridge reactor 240 (length = 63 mm, internal ∅ = 10 mm, V = 5 mL, see Figure S1, Supporting Information). The catalyst was loaded inside the cartridge (0.5 g, particle size: 315 μm–800 μm, bed length = 1.20 cm) together with glass wool and glass beads (d = 0.75 mm). The bromobenzene, phenylboronic acid, and toluene (internal standard) containing ethanol/H_2_O solution (v/v = 5/3) was pumped by a Knauer K‐501 HPLC pump with a flow speed of 0.1 mL min^−1^. Gas flow was regulated with a mass flow controller (Bronkhorst). The argon or oxygen gas flow rate was set as 0.2 mL min^−1^. Gas and liquid phase were mixed in a slit‐plate micro mixer (model LH2) before feeding the reaction mixture into the cartridge reactor. The temperature was set to 30 °C. The temperature of the reaction mixture was measured inside the cartridge reactor directly before the gas liquid mixture enters the cartridge. The pressure was monitored by a pressure sensor which was placed in front of the slit‐plate mixer. Samples were taken every 1 h for GC analysis.

##### Catalyst Characterization

X‐ray photoelectron spectroscopy (XPS) measurements were performed on an ESCALAB 220iXL (Thermo Fisher Scientific) with monochromated Al Kα radiation (E = 1486.6 eV). Samples are prepared on a stainless‐steel holder with conductive double‐sided adhesive carbon tape. The electron binding energies were obtained with charge compensation using a flood electron source and referenced to the C 1s core level of carbon at 284.8 eV (C—C and C−H bonds). For quantitative analysis, the peaks were deconvoluted with Gaussian–Lorentzian curves using the software Unifit 2023. The peak areas were normalized by the transmission function of the spectrometer and the element specific sensitivity factor of Scofield.^[^ ^37^ ^]^

Ex situ XAS measurements were performed at CAT‐ACT beamline at KIT Light Source.^[^ ^38^ ^]^ For ex situ measurements, all samples were prepared in form of pellets, suitable for the XAS measurements at the Pd K edge (24350 eV). XANES spectra and EXAFS data at the Pd K‐edge (DCM Si(311), collimated mirror Pt, focusing mirror Rh, beam size 2 × 2 mm^2^) were recorded in transmission mode (using ionization chambers filled with Ar, OKEN, Japan). EXAFS data up to k = 16 Å^−1^ were recorded at room temperature. All data were processed using the IFEFFIT package for background correction and normalization.^[^ ^39^ ^]^ The k^3^‐weighted EXAFS data at the Pd K‐absorption‐edges were Fourier transformed in the range of 3 to 12 Å^−1^ with the use of a Hanning window with a sill width of 1 Å^−1^. The EXAFS data for the Pd/PCN samples were fitted to the PdN reference (Materials Projects database, ID: mp‐999 293) using the Artemis package of IFEFFIT. For determining the structural parameters (coordination number and distances), fittings were performed in R‐space in the range from 1 to 4 Å; the amplitude (S_0_^2^) was set to 0.71 for all the samples, based on the analysis of PdO sample. The wavelet transform (WT) was conducted using the HAMA Fortran package. In a typical WT analysis, the parameters were set as *k*_*weigh*t_ = 0, utilizing the Morlet function with *κ* = 10 and *σ* = 1.

Powder X‐ray diffraction (XRD) patterns were recorded on a Panalytical X’Pert diffractometer equipped with a Xcelerator detector using automatic divergence slits and Cu Kα1/α2 radiation (40 kV, 40 mA; *λ* = 0.15406, 0.154443 nm). Cu beta‐radiation was excluded using a nickel filter foil. The measurements were performed with 0.087°s^−1^. The samples were mounted on silicon zero background holders. The obtained intensities were converted from automatic to fixed divergence slits (0.25°) for further analysis. Peak positions and profile were fitted with Pseudo‐Voigt function using the HighScore Plus software package (Panalytical). Phase identification was done by using the PDF‐2 database of the International Center of Diffraction Data (ICDD).

Scanning transmission electron microscopy (STEM) analysis was performed at 200 kV with an aberration‐corrected JEM‐ARM200F (JEOL, corrector: CEOS). The microscope is additionally equipped with a JED‐2300 energy‐dispersive X‐ray spectrometer (JEOL) having a silicon dry drift detector (dry SD60GV). For general imaging, high‐angle annular dark field (HAADF) and annular bright field (ABF) detectors were used. The solid powders were deposited without any pretreatment on a holey carbon‐supported Cu grid (mesh 300), which was then transferred into the microscope.

UV‐Vis diffuse reflectance spectra (UV‐Vis DRS) measurements of different materials were measured on a UV‐Vis‐NIR diffuse reflectance spectrometer (LAMBDA 365 UV/Vis Spectrophotometer) with a photometric range of 200–1000 nm at room temperature. Powders were prepared in a sample carrier with a quartz glass window at the edge of the integrating sphere with BaSO_4_ as the optical standard.

In situ diffuse reflectance infrared Fourier transform spectroscopy (DRIFTS) of CO adsorption and oxidation experiments were acquired with a Nicolet iS50 FTIR Spectrometer equipped with a mercury cadmium telluride (MCT) detector with a resolution of 4 cm^−1^ using 200 scans. An InProcess Instruments GAM 400 Quadrupole Mass Spectrometer (QMS) from Pfeiffer Vacuum with a Secondary Electron Multiplier was connected to monitor the outlet flow composition. Before CO adsorption, the sample was in situ pretreated in a flow (30 sccm) of Ar at 80 °C for 30 min and then cooled to 25 °C. The following tests were also conducted at 25 °C with the steady‐state mode. Next, the sample was exposed to 1% CO in Ar at a flow rate of 30 sccm for 30 min to reach the CO saturation coverage. Then, we purged the sample with 2 sccm Ar for 1 h to remove the gas phase CO from the cell, and a series of DRIFT spectra were collected during both the CO‐adsorption and the Ar‐purge process. The spectra were obtained by subtracting the background from the spectra of the samples after Ar pretreatment.

In situ ATR‐FTIR experiments were recorded using a setup consisting of a commercial high throughput and variable angle horizontal ATR accessory (Pike Technologies, ATRMAX II), which was mounted in a sample chamber of a Nicolet iS50 FTIR Spectrometer equipped with a liquid‐nitrogen cooled MCT detector. The attached ATR crystal is a trapezoidal ZnSe internal reflection element (45°, 56 × 10 × 4 mm^−3^, Pike Technologies). All ATR‐FTIR spectra were obtained by averaging 100 scans at 4 cm^−1^ resolution. All experiments were carried out at room temperature (296 K) in air. Solution flow was regulated by a compact dual piston pump (Knauer, Azura P4.1S), and all flow rates used in the experiments were 2 mL min^−1^.

In the substrate calibration, the background spectrum was collected with the solvent ethanol/H_2_O solution (v/v = 2/1). Then, the liquid phase was changed to the solution containing one or two substrates [either bromobenzene (1 mmol), iodobenzene (1 mmol), phenylboronic acid (2 mmol), or K_2_CO_3_ (2 mmol)] in 15 mL of ethanol/H_2_O as solvent (v/v = 2/1), and the spectrum was recorded. No catalysts were required in these experiments.

In the measurement with catalyst in solution (see Figure S2, Supporting Information), the background spectrum was collected with the reaction solution [bromobenzene (1 mmol), phenylboronic acid (2 mmol), and K_2_CO_3_ (2 mmol) in a 15 mL ethanol/H_2_O solution (v/v = 2/1)]. Then, the catalyst was added to the beaker, and a filter was used to separate the catalyst from the solution. With the filter, only the reaction solution is able to pass through the flow cell with a 45° ZnSe crystal (Pike Technologies, ATRMAX, 56×10×4 mm), while the Pd catalyst remains in the beaker. This allows for monitoring the product formation by recording the changing spectra of the reaction solution.

## Conflict of Interest

The authors declare no Conflict of Interest.

## Supporting information

Supplementary Material

## Data Availability

The data that support the findings of this study are available from the corresponding author upon reasonable request.
