# Supplementary material for: Atomically Dispersed Palladium Promoted Suzuki–Miyaura Cross Coupling
Source: ChemSusChem. 2025 Sep 10;18(20):e202500953. doi: 10.1002/cssc.202500953 (PMC12548944; doi:10.1002/cssc.202500953)
Supplement: Supplementary file 1 — Supplementary Material [file CSSC-18-e202500953-s001.pdf]

Supporting Information for

Atomically Dispersed Palladium Promoted Suzuki-Miyaura Cross-coupling

Junhao Huang[1], Marcus Klahn[1], Stephan Bartling[1], Anna Zimina[2], Nils Rockstroh[1], Norbert Steinfeldt[1], Tim Peppel[1], Jan-Dierk Grunwaldt[2], Jennifer Strunk[1, 3]*

[1] Leibniz Institute for Catalysis e.V., Albert-Einstein-Straße 29a, 18059 Rostock, Germany

[2] Institute of Catalysis Research and Technology and Institute for Chemical Technology and Polymer Chemistry, Karlsruhe Institute of Technology (KIT), 76131 Karlsruhe, Germany

[3] Industrial Chemistry and Heterogeneous Catalysis, Technical University of Munich, Lichtenbergstraße 4, 85748 Garching, Germany

*Corresponding author: jennifer.strunk@tum.de

This file includes:

Supplementary Note

Figures S1 to S11

Tables S1 to S7

References

Supplementary Note: **Discussion of experimental and computational methods for identification of reaction intermediates**

The transient nature of reaction intermediates make it challenging to capture and identify their exact composition and structure. To date, multiple specialist techniques based on traditional methods have been developed and applied for the identification of reaction intermediate. For example, Reimann et al.[1] utilized quick-scanning extended X-ray absorption fine structure (QEXAFS) spectroscopy to gain insight into the reaction mechanism of C–C coupling. Their study revealed the formation of colloidal Pd^0 clusters in the liquid phase and the reduction of supported Pd species in the solid catalyst. The formation of bromo-palladates ($[PdBr_4]^{2-}$, $[Pd_2Br_6]^{2-}$) was also observed. Denmark et al.[2] employed rapid injection NMR (RI-NMR) at low temperatures (e.g., -30 °C) to detect highly reactive intermediates. Canary et al.[3] reported the use of electrospray ionization mass spectrometry (ESI-MS) to observe transient catalytic intermediates involved in the Pd(0)-catalyzed coupling reaction of arylboronic acids with bromopyridines. It is worth noting that advances in characterization technique continue to enhance our ability to detect such transient species. For instance, by rationally controlling the ATR-FTIR measurement conditions, such as employing an *in situ* cell with rapid cooling and using a fast IR technique with data acquisition at the seconds or milliseconds level, these transient intermediates may become detectable, which would help differentiate between the reaction pathways.

Computational and kinetic studies have also made significant contributions to the understanding of reaction mechanisms. Maseras et al.[4] employed density functional theory (DFT) calculations to investigate the role of the base in the transmetalation step. Their findings suggest that the transmetalation step begins with the interaction between the base and the organoboronic acid. Additionally, extensive kinetic studies from Smith,[5] Soderquist,[6] Amatore and Jutand,[7] and Hartwig[8] have provided valuable insights into the role of the base and helped elucidate the dominant pathway involved in the transmetalation process.

However, although each method (QEXAFS, ESI-MS, rapid injection NMR, ATR-IR, DFT calculation, and kinetic study) has provided independent evidence for these reaction intermediates, the actual reaction pathway remains unresolved. Given the complexity of the reaction system (solvents, substrates, base, and catalysts), the combination of multiple analytical methods may offer a promising strategy for identifying and confirming the reaction mechanism and differentiate between the reaction pathways.

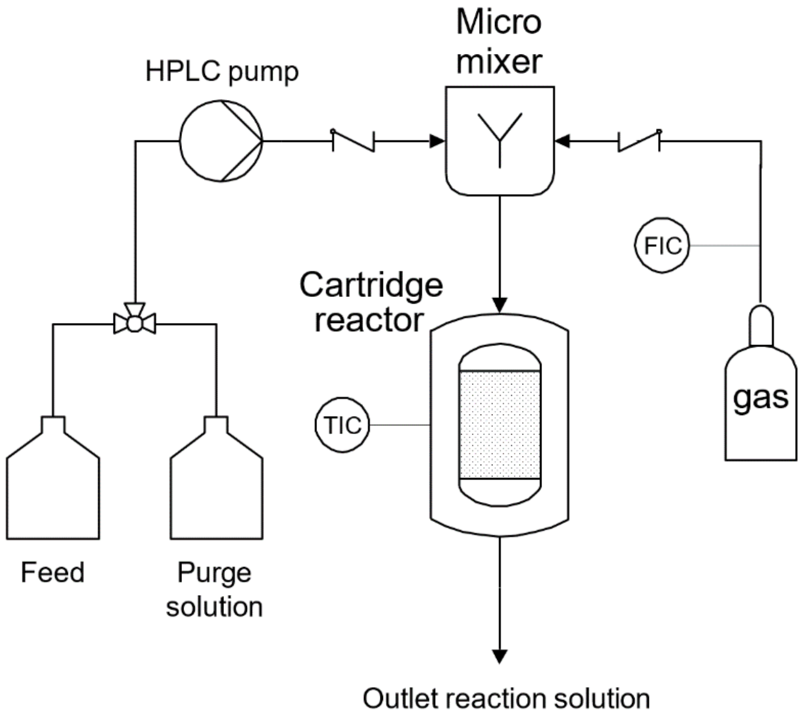

Figure S1. Schematic representation of the continuous flow reactor set-up used for Suzuki-Miyaura reaction.

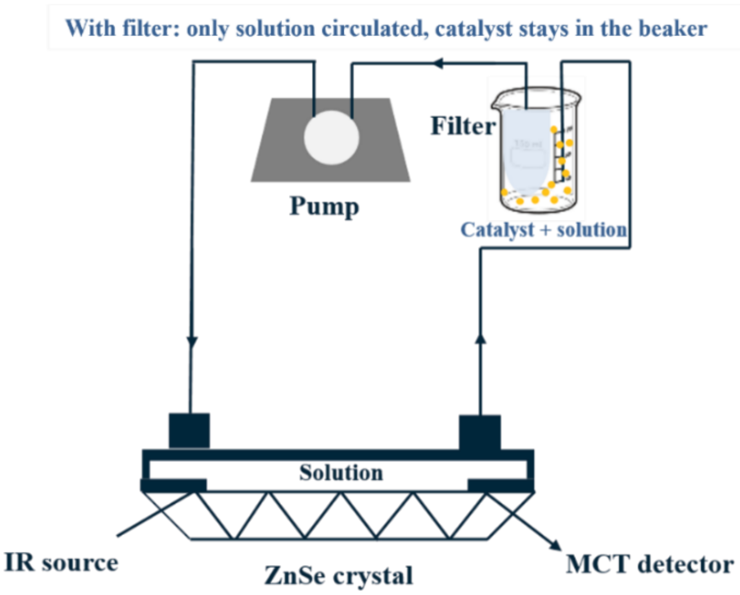

Figure S2. A schematic representation of the ATR set-up used to monitor the coupling of bromobenzene with phenylboronic acid over 2 wt% Pd/PCN catalyst.

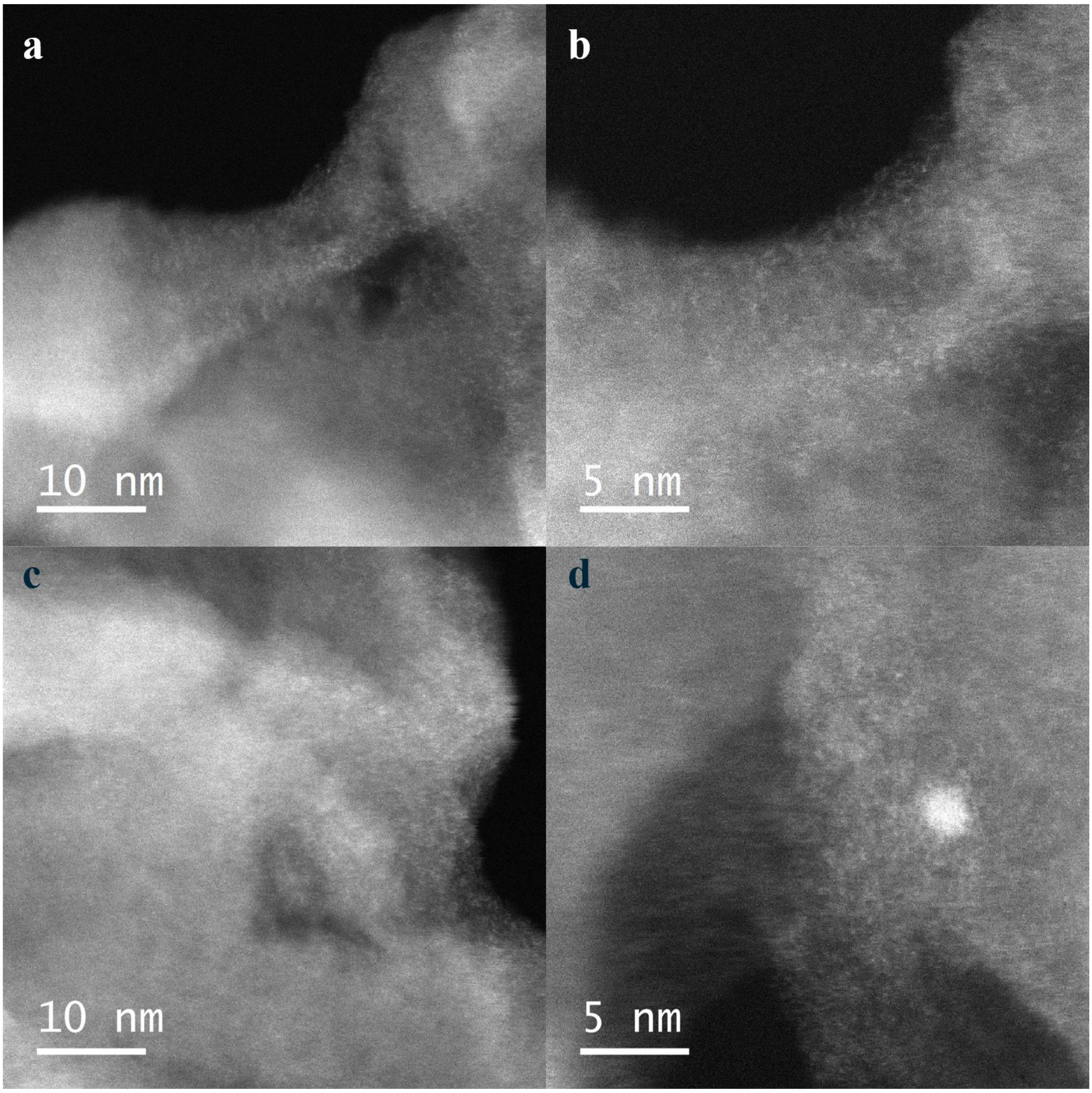

Figure S3. Representative HAADF-STEM images of 2 wt% Pd/PCN-200 °C. Please note that some decomposition of the PCN was observed in images *a* and *c* due to beam lability of PCN at high electron doses common at higher magnifications.

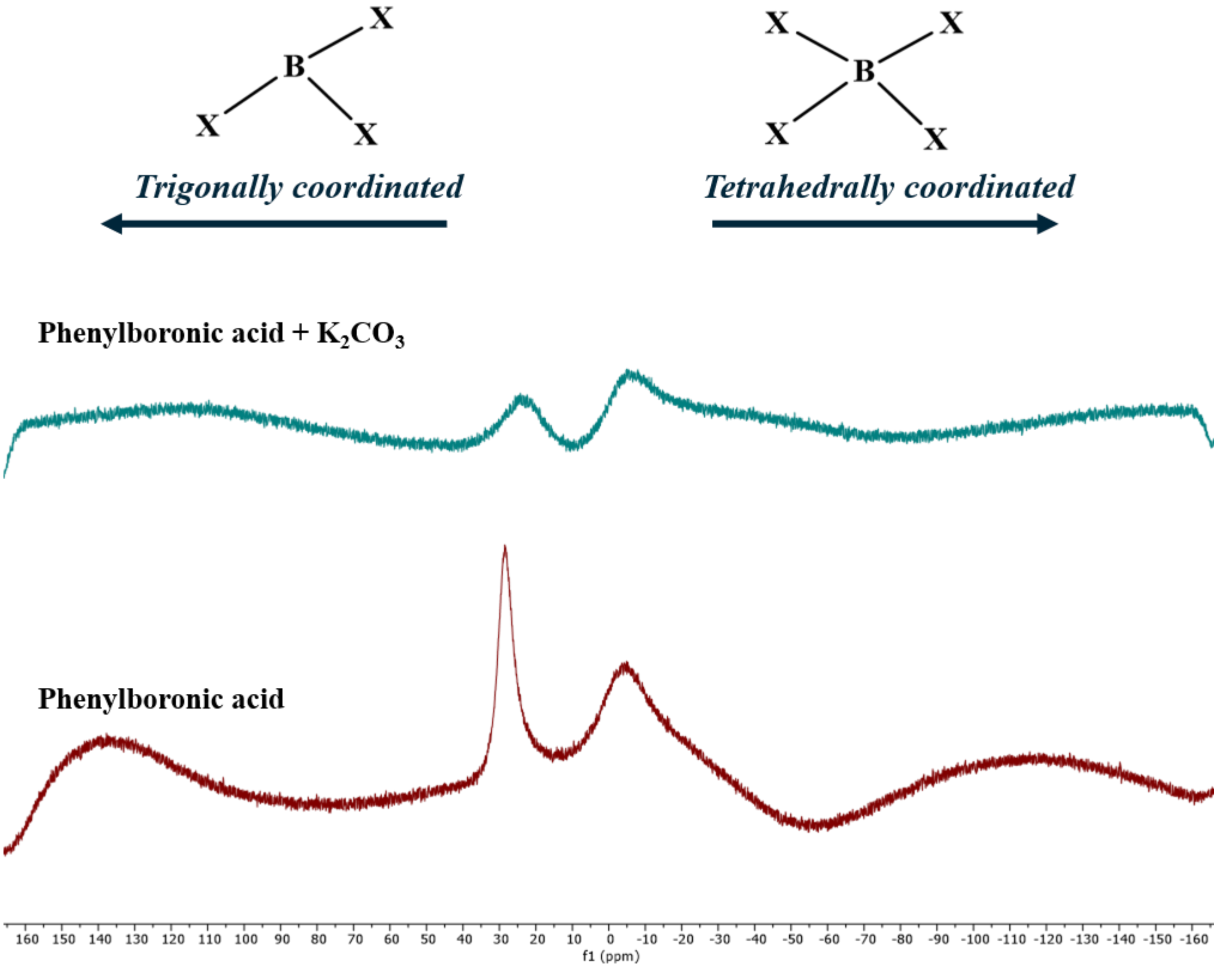

Figure S4. ^{11}B NMR spectra of phenylboronic acid with (green line) and without (red line) K_2CO_3. Note: Regular NMR tubes are made of borosilicate glass, which contains boron. Consequently, there are broad signals in the spectrum arising from the tube.

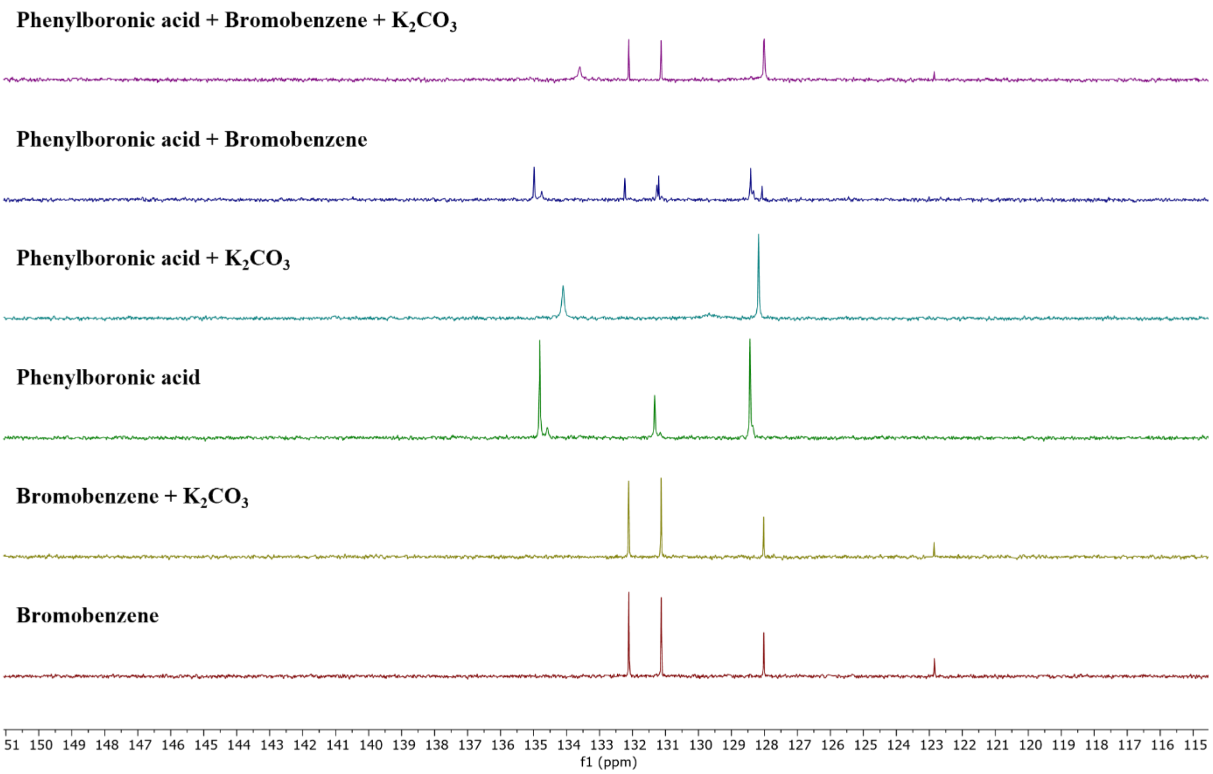

Figure S5. ^{13}C NMR spectra of substrates, referenced to Ethanol-d$_1$ (58.05 ppm).

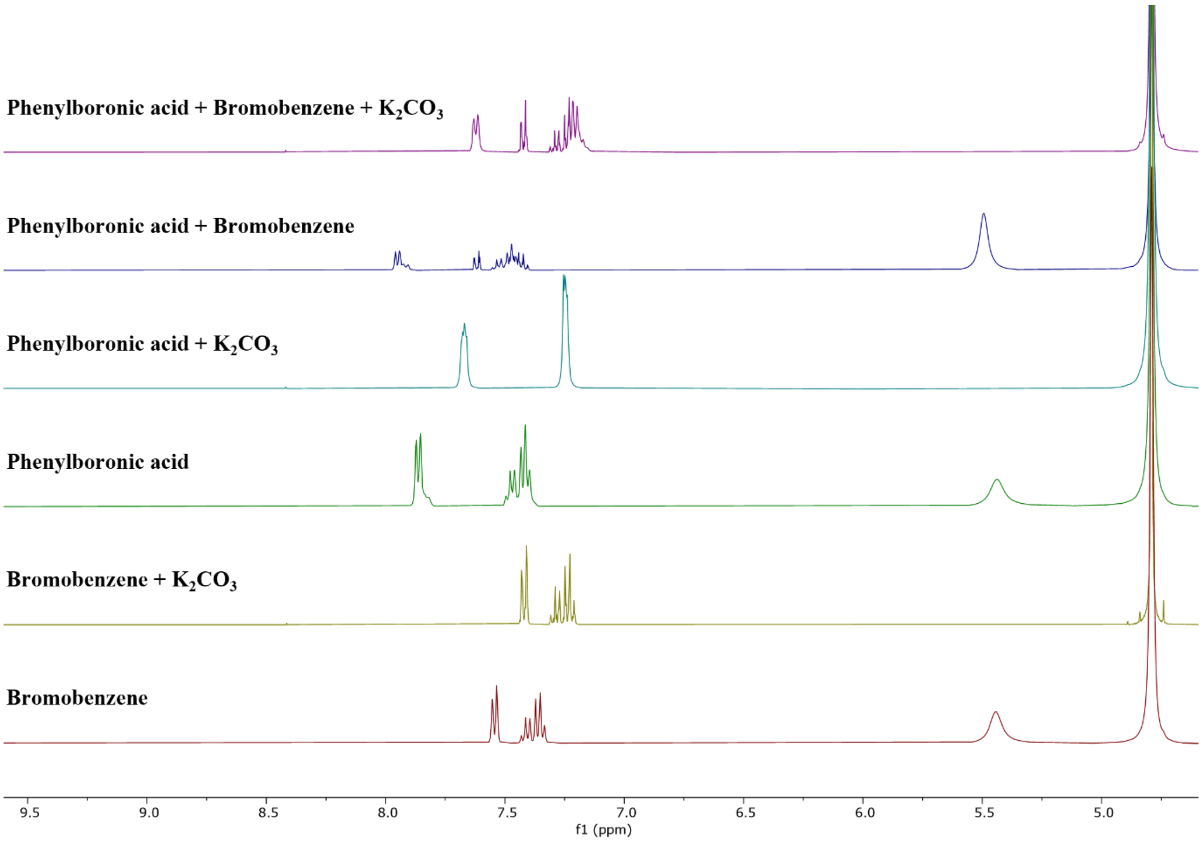

Figure S6. ^{1}H NMR spectra of substrates, referenced to D_2O (4.79 ppm).

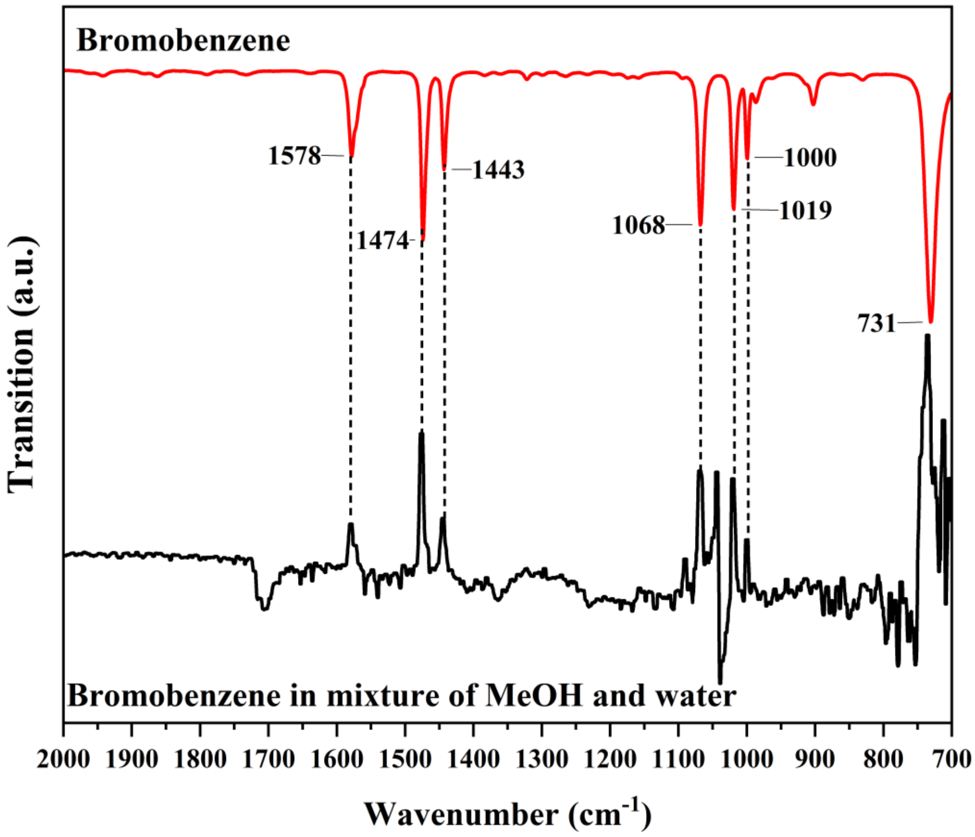

Figure S7. ATR-FTIR spectra of bromobenzene in pure form (red line) and dissolved in solvent (black line).

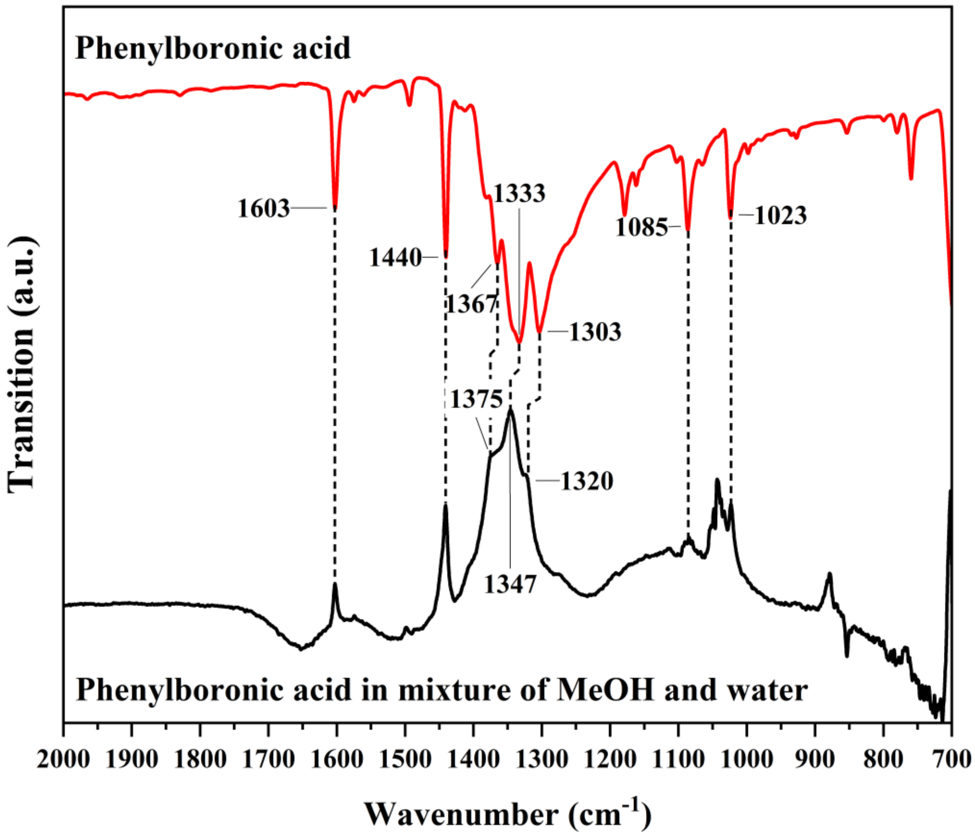

Figure S8. ATR-FTIR spectra of phenylboronic acid in pure form (red line) and dissolved in solvent (black line).

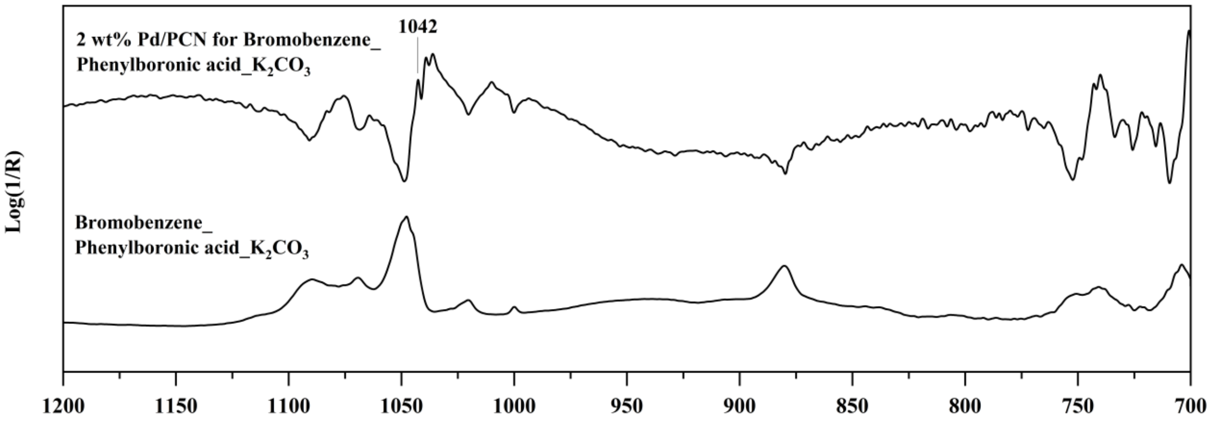

Figure S9. Enlarged ATR-FTIR spectra of 2 wt% Pd/PCN for the cross-coupling of bromobenzene and phenylboronic acid.

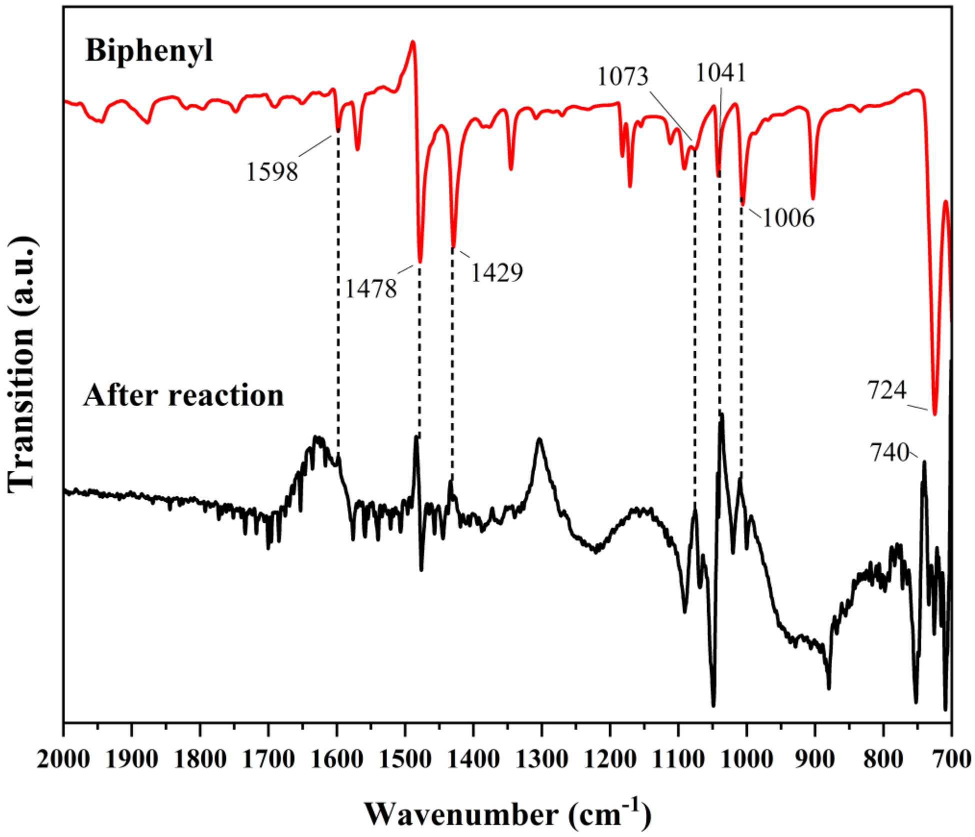

Figure S10. ATR-FTIR spectra of biphenyl in pure form (red line) and dissolved in solvent (black line).

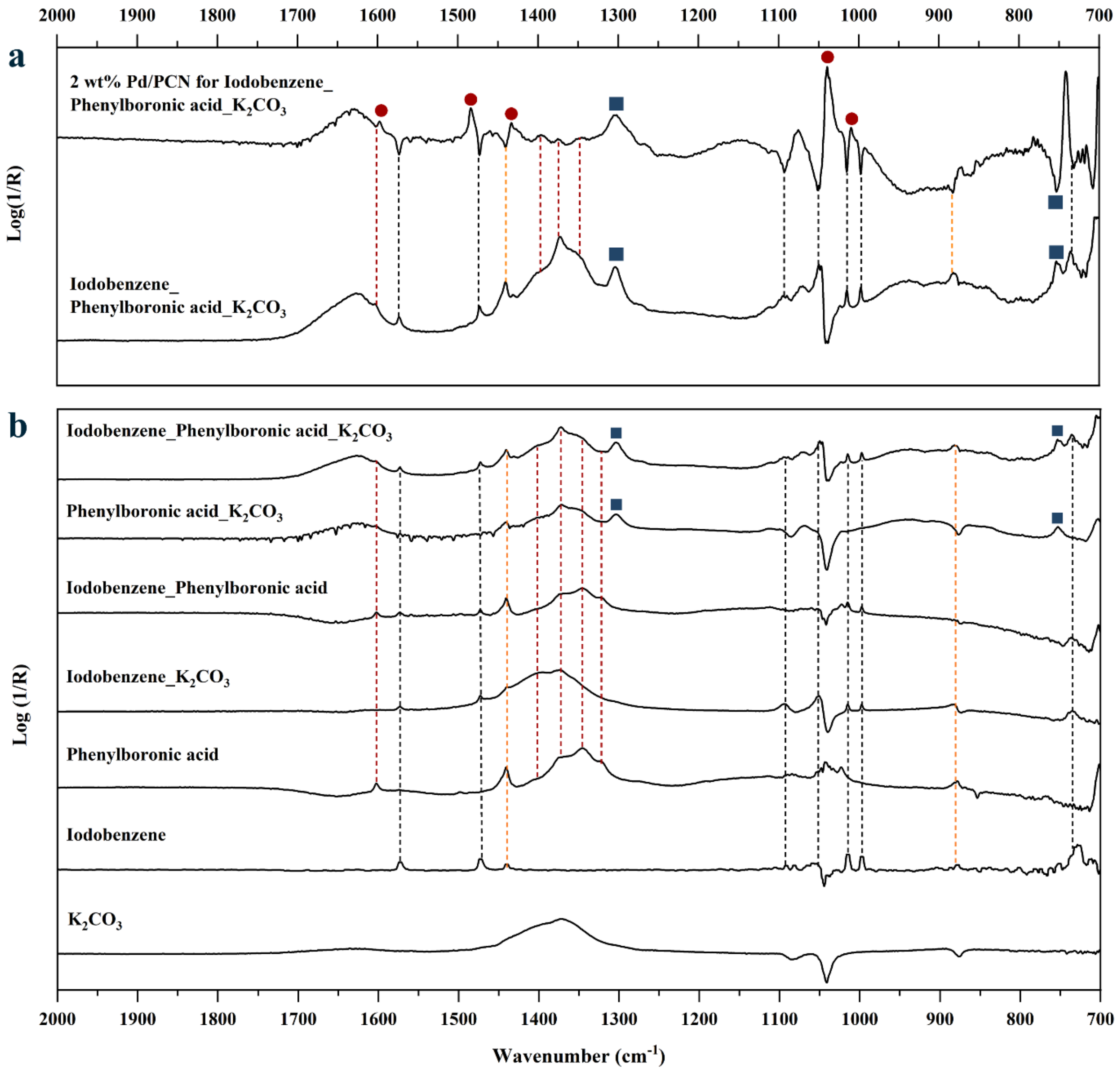

Figure S11. (a) *In situ* ATR-FTIR spectra of 2wt% Pd/PCN for the coupling reaction of iodobenzene and phenylboronic acid. (b) *In situ* ATR-FTIR spectra of the substrate calibration (iodobenzene, phenylboronic acid, and K_2CO_3). The bands related to the functional groups from bromobenzene and phenylboronic acid are marked with black and red dashed lines, respectively. The yellow dashed lines mark the IR bands present in both substrates. Newly emerged bands derived from boron-related group are marked with dark blue squares, and new bands related to biphenyl are marked with dark red spheres.

Table S1. Catalytic performance of various Pd/PCN catalysts, commercial 5% Pd/Al_2O_3 and 20% Pd/C catalysts.

Entry	Sample	Reactor	Atmosphere	TON	TOF (s^{-1})
1	0.5 wt% Pd/PCN	Batch	Argon	1118	0.31
2	1 wt% Pd/PCN	Batch	Argon	1267	0.35
3	2 wt% Pd/PCN	Batch	Argon	1732	0.48
4	4 wt% Pd/PCN	Batch	Argon	1327	0.37
5	8 wt% Pd/PCN	Batch	Argon	1226	0.34
6	16 wt% Pd/PCN	Batch	Argon	812	0.23
7	5% Pd/Al_2O_3	Batch	Argon	0	0
8	20% Pd/C	Batch	Argon	0	0

Table S2. EXAFS fitting parameters at the Pd K-edge for various Pd/PCN samples ($S_0^2 = 0.71$).

Sample	Shell	N	R (Å)	σ^2 (*10^{-3} Å^2)	ΔE (eV)	R-factor
Pd foil	Pd–Pd	12*	2.74 ± 0.01	4.7 ± 0.3	-4.2 ± 0.4	0.0034
PdO	Pd–O	4*	2.02 ± 0.01	1.0 ± 0.5	1.5 ± 1.0	0.0181
	Pd–Pd	4*	3.04 ± 0.01	8.1 ± 1.1		
	Pd–Pd	8*	3.43 ± 0.02	13.4 ± 1.9		
2 wt% Pd/PCN	Pd–N	4.2 ± 0.3	2.04 ± 0.01	2.6 ± 1.1	5.0 ± 0.7	0.0059
2 wt% Pd/PCN-200 ºC	Pd–N	3.4 ± 0.3	2.03 ± 0.01	1.2 ± 1.3	2.1 ± 1.0	0.0182
	Pd–Pd	3.2 ± 1.9	2.45 ± 0.03	19.0 ± 8.5		
2 wt% Pd/PCN-400 ºC	Pd–N	2.8 ± 0.3	2.03 ± 0.02	1.2	6.2 ± 1.4	0.0183
	Pd–Pd	7.7 ± 5.9	2.49 ± 0.04	29.7		
	Pd–Pd	0.6 ± 0.9	2.77 ± 0.02	0.4		

S_0^2 is the amplitude reduction factor, and S_0^2 was fixed to 0.71 as determined from PdO fitting; N is the coordination number; R is interatomic distance (the bond length between central atoms and surrounding coordination atoms); σ^2 is Debye–Waller factor (a measure of thermal and static disorder in absorber-scatterer distances); ΔE is edge-energy shift (the difference between the zero kinetic energy value of the sample and that of the theoretical model). R-factor is used to value the goodness of the fitting. * These values were fixed during EXAFS fitting, based on the known structure.

Table S3. High performance liquid chromatography (HPLC) data of 2 wt% Pd/PCN for continuous flow reaction in Ar and O_2 atmosphere at 0 hour, 4 hours, and 29 hours.

Reaction Atmosphere	Substrate	Retention time	Peak area					Catalytic property
			0 hour	4 hours*	Conversion[Δ]	29 hours*	Conversion[Δ]	
Ar	Phenylboronic acid	1.84	4.28E+04	3.65E+04	15%	4.13E+04	3%	Strong cross-coupling of bromobenzene and phenylboronic acid + Weak self-coupling of phenylboronic acid
	Toluene	7.58	5.05E+04	5.05E+04	-	5.05E+04	-	
	Bromobenzene	8.78	5.05E+04	3.18E+04	37%	4.48E+04	11%	
	Biphenyl	20.05	4.42	9.87E+04	-	5.08E+04	-	
O_2	Phenylboronic acid	1.84	3.55E+04	2.64E+04	26%	2.71E+04	24%	Weak cross coupling of bromobenzene and phenylboronic acid + Strong self-coupling of phenylboronic acid
	Toluene	7.58	4.39E+04	4.39E+04	-	4.39E+04	-	
	Bromobenzene	8.78	4.28E+04	3.90E+04	9%	4.23E+04	1%	
	Biphenyl	20.05	0	7.55E+04	-	5.03E+04	-	

* The peak areas of two substrates and product at reaction time t were recalculated according to the ratio of the toluene peak areas at reaction times 0 and t.

Δ The conversion was calculated using the equation: $CR = (S_0 - S_t) / S_0$, where CR is the conversion rate, S_0 and S_t are the peak areas of the specific substrate at reaction time 0 and t, respectively.

Table S4. Continuous flow experiments of 2 wt% Pd/PCN catalysts under argon and oxygen atmospheres.

Atmosphere	Sample	Real Time	TON*
Argon	2 wt% Pd/PCN	2 h	1555
		9 h	929
		29 h	536
Oxygen	2 wt% Pd/PCN	2 h	1501
		9 h	784
		29 h	452

* TON is calculated based on real-time biphenyl production collected at time t.

Table S5. XPS and ICP data of spent catalysts.

Sample	Metal content from XPS		Metal content from ICP	
	**Pd (at.%)**	**K (at.%)**	**Pd (wt%)**	**K (wt%)**
2 wt% Pd/PCN	1.1	0	2.02	0
2 wt% Pd/PCN_Ar	0.6	1.8	1.97	1.76
2 wt% Pd/PCN_O_2	0.6	1.8	1.98	2.40

Table S6. Catalytic performance of 2 wt% Pd/PCN, 2wt% Pd/PCN-200 °C, and 2 wt% Pd/PCN-400 °C.

Entry	Sample	Reactor	Atmosphere	Light	TON	TOF (s^{-1})
1	2 wt% Pd/PCN	Batch	Argon	-	1732	0.48
2	2 wt% Pd/PCN	Batch	Argon	+	1301	0.36
3	2 wt% Pd/PCN	Batch	Air	-	2323	0.65
4	2 wt% Pd/PCN	Batch	Air	+	1399	0.39
5	2 wt% Pd/PCN-200 ºC	Batch	Argon	-	57	0.02
6	2 wt% Pd/PCN-200 ºC	Batch	Argon	+	838	0.23
7	2 wt% Pd/PCN-200 ºC	Batch	Air	-	92	0.03
8	2 wt% Pd/PCN-200 ºC	Batch	Air	+	947	0.26
9	2 wt% Pd/PCN-400 ºC	Batch	Argon	-	0	0
10	2 wt% Pd/PCN-400 ºC	Batch	Argon	+	85	0.02
11	2 wt% Pd/PCN-400 ºC	Batch	Air	-	0	0
12	2 wt% Pd/PCN-400 ºC	Batch	Air	+	195	0.05

"+" and "–" indicate experiments conducted with and without light irradiation, respectively.

Table S7. Band frequencies and functional group assignments

Band frequency (cm^{-1})		Functional group
1625 - 1430	1603 (Phenylboronic acid)<br>1598 (Newly emerged band)<br>1576 (Bromobenzene)<br>1484 (Newly emerged band)<br>1476 (Bromobenzene)<br>1445 (Bromobenzene and Phenylboronic acid)<br>1433 (Newly emerged band)	Ring C=C stretching vibration
1450-1325		Asymmetric stretching vibrations of the CO_3^{2-}
1380 -1310		B−O stretching
1304 (Newly emerged band)		B−O stretching
1090, 1077, 1047, 1039, 1036, 1020, 1010, 1000		C−H in-plane vibration
1069		Br−Ar<br>(substituted aromatic ring and C−Br stretching combination)
1043 (Newly emerged bands)		Probably B−Br stretching
880		C−H out-of-plane vibration<br>(monosubstituted benzenes, five adjacent hydrogen atoms)
780 - 710	752 (Newly emerged band)<br>740 (Bromobenzene)<br>700 (Phenylboronic acid)	C−H out-of-plane vibration<br>(monosubstituted benzenes, five adjacent hydrogen atoms)

References

[1] S. Reimann, J. Stotzel, R. Frahm, W. Kleist, J. D. Grunwaldt, A. Baiker, *Journal of the American Chemical Society* **2011**, *133*, 3921-3930.

[2] A. A. Thomas, S. E. Denmark, *Science* **2016**, *352,* 329-332.

[3] A. O. Aliprantis, J. W. Canary, *Journal of the American Chemical Society* **1994**, *116,* 6985-6986.

[4] A. A. C. Braga, N. H. Morgon, G. Ujaque, F. Maseras, *Journal of the American Chemical Society* **2005**, *127*, 9298-9307.

[5] G. B. Smith, G. C. Dezeny, D. L. Hughes, A. O. King, T. R. Verhoeven, *The Journal of Organic Chemistry* **1994**, *59*, 8151-8156.

[6] K. Matos, J. A. Soderquist, *The Journal of Organic Chemistry* **1998**, *63*, 461-470.

[7] C. Amatore, A. Jutand, G. Le Duc, *Chemistry – A European Journal* **2011**, *17,* 2492-2503.

[8] B. P. Carrow, J. F. Hartwig, *Journal of the American Chemical Society* **2011**, *133*, 2116-2119.
